# *Aedes aegypti* SNAP and a calcium transporter ATPase influence dengue virus dissemination

**DOI:** 10.1371/journal.pntd.0009442

**Published:** 2021-06-11

**Authors:** Alejandro Marin-Lopez, Junjun Jiang, Yuchen Wang, Yongguo Cao, Tyler MacNeil, Andrew K. Hastings, Erol Fikrig

**Affiliations:** 1 Section of Infectious Diseases, Department of Internal Medicine, Yale University School of Medicine, New Haven, Connecticut, United States of America; 2 School of Public Health, Guangxi Medical University, Nanning, Guangxi, China; 3 State Key Laboratory of Virology, College of Life Science, Wuhan University, Wuhan, Hubei, China; 4 Department of Clinical Veterinary Medicine, and Key Laboratory for Zoonosis Research, Ministry of Education, College of Veterinary Medicine, Jilin University, Changchun, China; 5 Howard Hughes Medical Institute, Chevy Chase, Maryland, United States of America; Universidade Federal de Minas Gerais, BRAZIL

## Abstract

Dengue virus (DENV) is a flavivirus that causes marked human morbidity and mortality worldwide, and is transmitted to humans by *Aedes aegypti* mosquitoes. Habitat expansion of *Aedes*, mainly due to climate change and increasing overlap between urban and wild habitats, places nearly half of the world’s population at risk for DENV infection. After a bloodmeal from a DENV-infected host, the virus enters the mosquito midgut. Next, the virus migrates to, and replicates in, other tissues, like salivary glands. Successful viral transmission occurs when the infected mosquito takes another blood meal on a susceptible host and DENV is released from the salivary gland via saliva into the skin. During viral dissemination in the mosquito and transmission to a new mammalian host, DENV interacts with a variety of vector proteins, which are uniquely important during each phase of the viral cycle. Our study focuses on the interaction between DENV particles and protein components in the *A*. *aegypti* vector. We performed a mass spectrometry assay where we identified a set of *A*. *aegypti* salivary gland proteins which potentially interact with the DENV virion. Using dsRNA to silence gene expression, we analyzed the role of these proteins in viral infectivity. Two of these candidates, a synaptosomal-associated protein (AeSNAP) and a calcium transporter ATPase (ATPase) appear to play a role in viral replication both *in vitro* and *in vivo*, observing a ubiquitous expression of these proteins in the mosquito. These findings suggest that AeSNAP plays a protective role during DENV infection of mosquitoes and that ATPase protein is required for DENV during amplification within the vector.

## Author summary

Dengue is a mosquito-borne viral disease whose incidence has increased dramatically (>30-fold) in recent years, alongside the geographical expansion of *Aedes* mosquitoes. Dengue is currently one of the most important neglected tropical diseases, and its transmission is strongly influenced by rainfall, temperature, urbanization and distribution of the principal mosquito vector *Aedes aegypti*. Vector control strategies are being implemented to reduce the expansion of this disease, but still there is a great need for better therapeutics and preventive vaccines against dengue and other flaviviruses. Flaviviruses create complex virus-host and virus-vector interactions. The interactions between viral particles and protein components in the vector are not completely understood. In this work we characterize how two mosquito proteins, “AeSNAP” and “ATPase”, influence DENV viral dissemination within *A*. *aegypti*, using both *in vitro* and *in vivo* models. These results suggest that anti-vector measures that target specific mosquito molecules may represent promising alternatives to traditional countermeasures against dengue virus spread.

## Introduction

Dengue is a major public health threat in tropical and subtropical areas, and as climate change and urbanization continues, the illness may spread to other locations across the globe [1]. According to reports from the World Health Organization, before 1970 only nine countries experienced outbreaks of severe dengue. Today, the disease is endemic in more than 100 countries in Africa, the Americas, South-East Asia, the Western Pacific regions, and the Eastern Mediterranean regions. Recently, some dengue cases have been documented in places where the disease was absent for more than 50 years, including France and Spain (European Centre for Disease Prevention and Control). Therefore, more attention is required to develop countermeasures to address this expansion, fed by processes including global warming, unprecedented human mobility, rapid urban population growth, and large-scale changes in ecosystems [2–5].

Dengue virus (DENV) is a positive-sense, single-stranded RNA virus that belongs to the genus *Flavivirus* within the family *Flaviviridae*. Its primary vector is the *Aedes aegypti* mosquito. After taking a viremic blood meal, DENV establishes infection in the midgut. The midgut represents the first barrier to block viral propagation in the mosquito. Upon establishing a successful infection, the virus disseminates systemically through the hemolymph where it can invade secondary tissues, such as the salivary glands [6]. Replication in the salivary glands leads to virion release into the saliva, the last step prior to virus transmission to the human host [7].

Dengue is provoked by four serologically different DENV serotypes and usually results in a mild self-limiting disease, but is also capable of causing much more severe dengue hemorrhagic fever (DHF) or dengue shock syndrome (DSS) with approximately 20,000 fatalities recorded annually [8]. Conventional vaccines are in development and some of them are being implemented in a limited number of countries [9]. The development of these vaccines has been complicated by to the co-circulation of different serotypes and the phenomena of antibody-dependent enhancement (ADE) [10–13]. ADE occurs when an individual who has encountered one DENV serotype is infected by a second DENV serotype and non-neutralizing antibodies bind to the virus allowing it to enter mononuclear cells, susceptible to virus infection, via an FcR-dependent mechanism. This process leads to greatly enhanced disease severity [14]. Therefore, exploring novel methods to block DENV spread in the mosquito vector by analyzing ways to interfere with vector-virus interactions, could be a good alternative to, or complement for conventional vaccines. The targeting, modification, or elimination of specific genes in *A*. *aegypti* can reduce vector competence for virus acquisition, dissemination, and transmission [15–17], reducing the expansion of this widespread arboviral disease. Indeed, transmission-blocking vaccines (TBV) may trigger a strong immune response against mosquito components, which can block the viral infection in vector tissues [18]. This has been shown to be the case for C-type lectins and the cysteine rich venom protein CRVP379 in the mosquito. When the interaction between the virus and vector proteins are blocked using specific antibodies, DENV infection in *A*. *aegypti* is effectively interrupted [19,20].

DENV replication in the salivary gland is the last step before virus transmission to the mammalian host, and little is known about the protein interactions that take place at this stage. Here, we explore the impact of altering protein expression levels of several *A*. *aegypti* proteins found ubiquitously in mosquito tissues during DENV infection, *in vitro* and *in vivo*. Using viral purification coupled to a mass spectrometry assay, we identified a set of *A*. *aegypti* proteins which potentially interact with DENV virions. Next, we used dsRNA silencing to analyze the effect of these interaction candidates during DENV infection. Using these techniques, we demonstrate that a synaptosomal-associated protein with a T-Snare domain (AAEL005449), that we named here AeSNAP, and a calcium transporter ATPase protein (AAEL006582, ATPase) have a role in DENV infection *in vitro*, in the Aag2 *A*. *aegypti* cell line, and *in vivo* in the *A*. *aegypti* mosquito. Silencing of AeSNAP expression led to an increase in viral burden at 24 hour post-infection (hpi) *in vitro* and 7 dpi in the mosquito, whereas we found the opposite result after silencing ATPase protein expression. These findings suggest that AeSNAP may have a protective role during DENV infection whereas ATPase protein is required for DENV during amplification. This highlights two possible targets for controlling DENV transmission in the mosquito vector.

## Materials and methods

### Ethics statement

All experiments were performed in accordance with guidelines from the Guide for the Care and Use of Laboratory Animals (National Institutes of Health). The animal experimental protocols were approved by the Institutional Animal Care and Use Committee (IACUC) at the Yale University School of Medicine (assurance number A3230-01). All infection experiments were performed in an arthropod containment level 3 lab (ACL3) animal facility according to the regulations of Yale University. Every effort was made to minimize animal pain and distress.

### Cell culture and virus production

Two *Aedes spp*. cell lines were used in this study, Aag2 and C6/36 cells. The *A*. *aegypti* cell line, Aag2 (ATCC, VA), was used for *in vitro* silencing studies described below. Aag2 cells were grown at 30 °C with 5% CO_2_ in DMEM high glucose media supplemented with 10% heat-inactivated fetal bovine serum (Gibco), 1% penicillin-streptomycin. In addition, the *A*. *albopictus* cell line, C6/36, was used to grow DENV stocks using the same media. The dengue strain DENV-2 New Guinea C was used. C6/36 cells were infected at an MOI of 1.0. The culture supernatant was harvested 6 days after infection and subjected to a plaque assay to determine the viral titer, using BHK-21 clone 15 cell line (gently provided by Dr. Brackney, at the Connecticut Agricultural Station), grown at 37 °C in MEM supplemented as described. Virus stock was stored at −80 °C before use.

### Mosquitoes

*A*. *aegypti* (Orlando strain, obtained from the Connecticut Agricultural Experiment Station) mosquitoes were maintained on 10% sucrose feeders inside a 12- by 12- by 12-in. metal mesh cage (BioQuip; catalog no. 1450B) at 28°C and ∼80% humidity with a 14:10 h light:dark photoperiod. Egg masses were generated via blood meal feeding on naïve 129 mice. All mosquitoes were housed in a warm chamber in a space approved for BSL2 and ACL3 research. Mosquitoes were used in these experiments 2–14 days after emergence.

### Preparation of DENV and salivary gland mixture

Cell-free supernatants were taken from a T-150 flask of DENV2-infected C6/36 cells at 10 dpi and overlaid on top of room-temperature 30% sucrose-PBS. Samples were ultra-centrifuged at 100k x g for 2 hours and supernatant was removed before viral pellet was resuspended in ~2 mL serum-free DMEM media. Resuspended virus was overlaid on a room-temperature 30%/60% sucrose-PBS gradient and ultra-centrifuged at 100k x g for 2 hours. Using a flashlight shone from underneath the virus/sucrose-gradient, a viral band was visualized and ~600 μL was carefully pipetted to a new Eppendorf tube. A small aliquot was removed to determine viral titer. Salivary glands from *A*. *aegypti* mosquitoes (Orlando strain) were dissected. Briefly, 10–14 day old uninfected and sucrose-fed female mosquitoes were anesthetized on ice, followed by the removal of the legs and head. After pushing down the thorax, salivary gland was isolated by using small forceps, and placed into sterile phosphate-buffered saline (PBS) on ice. Sucrose-purified virus was split into two aliquots of ~300 μL (~3.9 x10^9^ viral particles in each) and extract from 10 *A*. *aegypti* salivary glands (SGE) in 10 μL was added to one of the aliquots. Both the DENV (control) and the DENV+SGE were incubated for 1 hour at 30°C, and then diluted to 4 mL before being overlaid on a room-temperature 30%/60% sucrose-PBS gradient and ultra-centrifuged at 100k x g for 2 hours. Both bands of the DENV (control) and the DENV+SGE were collected as described above and heat inactivated for 10 minutes at 65°C before being frozen at -80°C. This entire protocol was repeated for three biological replicates.

### Liquid chromatography and tandem mass spectrometry analysis (LC + MS/MS)

DENV (control) and DENV combined with salivary gland extract samples were submitted to the Interdisciplinary Center for Proteomics at the Yale University, where they were precipitated and resuspended in PBS before liquid chromatography tandem mass spectrometry (LC + MS/MS). Charge state deconvolution and deisotoping were not performed. All MS/MS samples were analyzed using Mascot. Mascot was set up to search the *Aedes aegypti*_201505 database (selected for *Aedes aegypti*, unknown version, 37,800 entries) assuming the digestion enzyme trypsin. Mascot was searched with a fragment ion mass tolerance of 0.050 Da and a parent ion tolerance of 10.0 PPM. Carbamidomethyl of cysteine was specified in Mascot as a fixed modification. Gln- > pyro-Glu of the n-terminus, deamidated of asparagine and glutamine and oxidation of methionine were specified in Mascot as variable modifications. Scaffold (version Scaffold_4.4.8, Proteome Software Inc., Portland, OR) was used to validate MS/MS based peptide and protein identifications. Peptide identifications were accepted if they could be established at greater than 50.0% probability by the Peptide Prophet algorithm with Scaffold delta-mass correction. Protein identifications were accepted if they could be established at greater than 50.0% probability and contained at least 1 identified peptide. Protein probabilities were assigned by the Protein Prophet algorithm. This entire protocol was repeated for three biological replicates. The mass spectrometry proteomics data have been deposited to the ProteomeXchange Consortium via the PRIDE [21] partner repository with the dataset identifier PXD024959.

### dsRNA production, silencing and DENV infection *in vitro*

For gene knockdown, dsRNA was produced from approximately 500 bp coding regions of either *A*. *Aegypti* candidate analyzed in this study or green fluorescent protein (GFP) as a control. Genes that were found in at least two out of the three independent experiments were selected for further studies (Table 1). Briefly, MS gene candidates were cloned in the pMT-BiP-His-V5 vector using cDNAs from Aag2 or salivary gland. PCR was used to produce a DNA template with T7 overhangs that was then used to generate the dsRNA molecules (TranscriptAid T7 High Yield Transcription Kit, ThermoScientific), according to manufacturer’s instructions. Oligos used for making dsRNA are shown in Table 2. For *in vitro* studies, the dsRNA molecules were transfected into Aag2 cells (INTERFERin, Polyplus) according to manufacturer’s instructions. Briefly, 500 ng of dsRNA were added to 5 x 10^5^ cells in a 48 well plate. 48 h post-transfection, silencing level was analyzed, cells were infected with DENV2 at MOI 1.0, and DENV2 viral burden was analyzed at different timepoints. No significant variations regarding survival were observed between groups.

**Table 1:** List of putative DENV binders obtained from the *A aegypti salivary* gland extract by mass spectrometry assay.

RUN 1/2/3	Acc number	Protein name/putative function	Abrev name
	AAEL012585	Ribonuclueoprotein, ribosomal protein L30 [RpL7]	Rpl7
	ABF18051.1	rRNA binding, 40S ribosomal protein S4	S4
	AAEL000068	40S Ribosomal protein S25	S25
	AAEL003743	V-type protein ATPase subunit a, hydrogen ion transmembrane transporter activity	Vtype
	AAEL004559	Synaptosomal-associated protein, T-Snare domain	AeSNAP
RUN 2/3			
	AAEL004538	Polypeptide N-acetylgalactosaminyltransferase, carbohydrate binding, transferase activity, transferring glycosil groups	Ricin
	AAEL008123	DNAdependent protein kinase activity, double strand break repair vi nonhomologous end joining	Break
	AAEL006917	MG-160, Golgi apparatus protei, E selectin ligand	MG160
	AAEL010146	Fatty acid beta oxidation, 3 hydroxyacil coA dehydrogenase activity, enol coA hydratase activity	Fatty
	AAEL005185	Leucin rich repeat protein	Leu
	AAEL013274	Polypeptide N-acetylgalactosaminyltransferase, carbohydrate binding, transferase activity, transferring glycosil groups	Ricin2
	AAEL002346	Semaphorin receptor activity	Semaphorin
	AAEL008642	DNAj protein, HSP40 protein	HSP40
RUN 1/3			
	AAEL009747	rRNA binding, ribosomal protein S18	S18
	AAEL006582	Calcium transporting ATP ase, ATP binding, metal ion binding	ATPase
RUN 1			
	AAEL001872	VDAC, voltage-gated anion channel activity	
RUN 2			
	CYP305A5 AAEL002043-PA	Cytochrome P450, heme binding, iron ion binding, oxidoreductase activity, acting on paired donors, with incorporation or reduction of molecular oxygen	
	A0A0P6K119	Putative flotilin, form membrane microdomains	
	AAEL014260	Zink-finger protein, nuceil acid binding, zinc ion binding	
	AAEL005458	Acyltransferase	
	AAEL012175	ATP synthase subunit alpha	
RUN 3			
	AAEL009993	Self proteolysis, salivary gland secreted protein domain toxin	
	AAEL009357	Myosin motor, ATP binding	
	AAEL006417	D7 protein, odorant binding	
	AAEL007080	O acyltransferase activity, cellular lipid metabolic process	
	AAEL004351	Kinase, serine/threonine protein kinase, ATP binding	
	A0A0P6KIVH7	beta N acetylhexosaminidase activity	
	A0A0P6IXE5	Putative dystrophin-like protein, acting binding, zinc ion binding	
	AAEL015065	Src homology 3 [SH3] domain, EF-hand calcium binding domain	
	AAEL011737	F box protein	
	CYP6N9	Cytochrome P450, heme binding, iron ion binding, oxidoreductase activity, acting on paired donors, with incorporation or reduction of molecular oxygen	
	AAEL005929	ABC transporter domain	
	AAEL005845	Phospholipid binding, structural constituent of cytoskeleton	
	AAEL005417	Annexin, calcium dependent phospholipid binding	
	AAEL006424	37kDa salivary gland allergen Aed a 2	
	AAEL013936	Serpin family protein	
	AAEL008099	Fe2+ 2 oxoglutarate dyoxigenase, nucleotide-diphosphosugar transferase	
	AAEL007525	EF Hand calcium binding domain	
	AAEL013170	O acyltransferase activity, cellular lipid metabolic process	
	AAEL012690	3–5 exonuclease activity, nucleic acid binding	
	AAEL009662	Nuclear prerribosomal associated protein 1	
	AAEL008138	ABC transporter domain	
	AAEL008144	Catalytic activity/AMP binding	
	AAEL000483	Acetylglucosaminyltransferase activity	

**Table 2:** List of primers used for cloning, RNA knockdown and qRT-PCR analysis.

Gene		Clone primer	dsRNA primer	qPCR primer
RPL7	Forward	CATGGCCCGG GGTACC T ATGCCAGCTGCGGCTAAG	TAA TAC GAC TCA CTA TAG GG GCCAGCTGCGGCTAAGACTG	GGTGTTGCAGTTGTTCCGTC
	Reverse	TAGACTCGA GCGGCCGC CA GA TCATACGCTGGATAAGCTCA	TAA TAC GAC TCA CTA TAG GG TGCGGACCTTGGGGGCGACC	GTAGGTGATGTACGGCTCGG
S4	Forward	CGGGAGATCT CCATGG ATGGCTCGCGGACCGAAGAA	TAA TAC GAC TCA CTA TAG GG ATGGCTCGCGGACCGAAGAA	CGAACCCGGAAACCTGTGTA
	Reverse	TAGACTCGA GCGGCCGC CA GTGAGCAGCCTTGCTGGCCAG	TAA TAC GAC TCA CTA TAG GG GGTCAGAATGAATGGCACCT	GCAGCGAGATGAAAGCCTTG
S25	Forward	CATGGCCCGG GGTACC T ATGCCTCCGAAGAAGGATACCA	TAA TAC GAC TCA CTA TAG GG ATGCCTCCGAAGAAGGATAC	CTGCGGGAACTTTGCCAGAA
	Reverse	TAGACTCGA GCGGCCGC CA TGCCACTGGATCGTCTCCCT	TAA TAC GAC TCA CTA TAG GG AACGTTCGGACACGACCGAG	TATGCCACTGGATCGTCTCC
V-type	Forward	CATGGCCCGG GGTACC T ATGGGTTCGCTGTTCCGCAGC	TAA TAC GAC TCA CTA TAG GG ATCGAAACCATCCAGATTGC	GGACTGTTCAACCACCGCTA
	Reverse	TAGACTCGA GCGGCCGC CA TTCTTCCGTAGAGCTGGAAC	TAA TAC GAC TCA CTA TAG GG ATAGGGCGTTCCCGAGTAGT	GATCAAAATGGACGGAGCGC
AeSNAP	Forward	CATGGCCCGG GGTACC T ATGCCTGCTGCAGTACCCGCA	TAA TAC GAC TCA CTA TAG GG GTACCCGCAGAGAATGGAGGCGG	ACATGGGCCAAGTGAACACA
	Reverse	TAGACTCGAGCGGCCGCCA GCTTCGTAACAGCTGACCAGT	TAA TAC GAC TCA CTA TAG GG ATGTAACCCGCTTGTGGTCC	TGCTGGCTTCTTCAAGTCGT
Ric	Forward	CATGGCCCGG GGTACC T ATGCGGGTAACCAACATCCG	TAA TAC GAC TCA CTA TAG GG ACGTTGCTTACGACGTGTTG	TGATCGAAGGACTGGGCAAC
	Reverse	TAGACTCGA GCGGCCGC CA CCACCGCGGAGTGATGGTG	TAA TAC GAC TCA CTA TAG GG AGTTCCACACCATCACTCCG	ATTTTGCTGATCCTCCGGCA
MG160	Forward	CATGGCCCGG GGTACC T ATGGAAGATGCGTTGCTTGG	TAA TAC GAC TCA CTA TAG GG GTTTCCGACGACAAGGATGT	ATCTGATCCCCGAGGTGGAA
	Reverse	TAGACTCGA GCGGCCGC CA CTTCTCGCAGTCCGCGTG	TAA TAC GAC TCA CTA TAG GG AGTTCCTGTAAAGCTGCGGA	AAAAGTGCAAAGACGCGGTC
Break	Forward	CATGGCCCGG GGTACC T CTGTACGTCATCGGGATACA	TAA TAC GAC TCA CTA TAG GG CTAACGCAACCGTCCAAAAT	ACCGTGAAAGCAGCGTAAGA
	Reverse	TAGACTCGA GCGGCCGC CA GAGTGCAAATGGACTAGCCA	TAA TAC GAC TCA CTA TAG GG AACACGACCTTCCTCACCAC	CTCCATACACTTGTGCCGGT
Fatty	Forward	CGGGAGATCT CCATGG ATGGCCAGCTTAAGACTGAT	TAA TAC GAC TCA CTA TAG GG ACAGCACTGGATCTGGCTCT	CCGGGATTCTACACGACTCG
	Reverse	TAGACTCGA GCGGCCGC CA CTTGCTCGGGTAAAACTTTTC	TAA TAC GAC TCA CTA TAG GG ACCTGAACGATACCAGCACC	GTGCGCACCAACATCGATAC
Leu	Forward	CATGGCCCGG GGTACC T ATGTCCAAACGAGTTGCAACA	TAA TAC GAC TCA CTA TAG GG CCATTGAAGCGTGCAACTGT	GCAGAGTAACGGTTTACTGCG
	Reverse	TAGACTCGAGCGGCCGCCA ATTATGGAAGAATATATTAAG	TAA TAC GAC TCA CTA TAG GG TCCGCATTTGGCTTCTCAGA	GTCGCCAACCAAGTCAGGAT
Ric2	Forward	CATGGCCCGG GGTACC T ATGCGGGTAACCAACATCCG	TAA TAC GAC TCA CTA TAG GG CTGATCGAAGGACTGGGCAA	GCGATGATCGGACTACAGCA
	Reverse	TAGACTCGA GCGGCCGC CA TTGATATGCATTGTGCACGTCG	TAA TAC GAC TCA CTA TAG GG ACGATAACTTCCCCGGTGGC	TGTTCTCGTCGTACTGCCAC
Sem	Forward	CGGGGTACCT ACTAGT TGCGATTGGTGCGTGGAAG	TAA TAC GAC TCA CTA TAG GG ACGTGTACGGAGGGATCAAG	CCAAGTTGGGCAGCAAGTTC
	Reverse	TAGACTCGA GCGGCCGC CA TTGGCCGATGTCGTACTCTA	TAA TAC GAC TCA CTA TAG GG GACGTAGTCAATCGACGGGT	CCGTCCGTTGATCGTCAGAT
HSP40	Forward	CGGGGTACCT ACTAGT AACTGCCGTCCATTGGTGGATC	TAA TAC GAC TCA CTA TAG GG CCGAACTGAAAGAGCTTTGG	GGCTCAGATGTACCATCCCG
	Reverse	TAGACTCGA GCGGCCGC CA AGAACAGTGCACAGTGTGGT	TAA TAC GAC TCA CTA TAG GG TGAGAGGTGGTTTCTTCGCT	CTTGGATTGGGCCCATCTGT
S18	Forward	CATGGCCCGG GGTACC T ATGTCGCTCGTGATCCCAGAG	TAA TAC GAC TCA CTA TAG GG TCGCTCGTGATCCCAGAGAA	CCTCCAACGTCGACTCCAAA
	Reverse	TAGACTCGA GCGGCCGC CA CTTCTTCTTGGACACACCGA	TAA TAC GAC TCA CTA TAG GG TCAGCTGCGAGTACTTGCCA	TCTTCTTGGACACACCGACG
ATP	Forward	CCGGGTACCT ACTAGT ATGGAGGACGGCCATAGCAA	TAA TAC GAC TCA CTA TAG GG AACCGCATCTTGGATCTGAC	GATGTCCGTCTCGCGTATGT
	Reverse	TAGACTCGA GCGGCCGC CA CTTGGCGACAGCGGTACCAG	TAA TAC GAC TCA CTA TAG GG AACGATCTTGGACTTGTGGG	GATGGTTCCCACTTCCTGCA
Rp49	Forward			GCTATGACAAGCTTGCCCCCA
	Reverse			TCATCAGCACCTCCAGCT
DENV	Forward			GCCAAAGTCACACACCCTCT
	Reverse			ACCTAGATGCCATGGTCCTG
GFP	Forward		TAA TAC GAC TCA CTA TAG GG ACGTAAACGGCCACAAGTTC	ACGTAAACGGCCACAAGTTC
	Reverse		TAA TAC GAC TCA CTA TAG GG TGTTCTGCTGGTAGTGGTCG	TGTTCTGCTGGTAGTGGTCG

Three different experiments of mass spectometry assays were performed, and hits were clustered according to their presence in every experiment. Accession numbers, putative functions and abbreviated names were shown.

Gene candidates found in at least 2 independent experiments were cloned in the plasmid vector. dsRNA molecules were generated for RNAi experiments and the gene expression was analyzed by qRT-PCR. qRT-primers were designed using Primer3plus and RNA knockdown primers were designed using SnapDragon—dsRNA Design, platform supported by DRSC/TRiP Functional Genomics Resources at Harvard Medical School.

### *In vivo* mosquito silencing and infection

For dissemination studies, 2–10 day old female mosquitoes were injected with dsRNA to silence the individual genes (n = 17–25 mosquitoes/group). As a control, mosquitoes were injected with dsRNA for GFP (dsGFP). Mosquitoes were kept on ice for 15 min, and then transferred to a cold tray to receive an intrathoracic microinjection via the lateral side of the thorax of 1 μg of dsRNA diluted in 138 nl of MQ water, using a Nanoinject II Injector (Drummond Scientific, USA). After injection, the mosquitoes were transferred into cylindrical containers fitted with a nylon mesh on the top and supplied with 10% sucrose solution. 72 hours post dsRNA injection, mosquitoes were infected with DENV2 through intrathoracic injection. Female mosquitoes were immobilized in a cold tray and intrathoracically inoculated with 100 PFU of DENV2 in 138 nl, as previously described. The infected mosquitoes were then dissected on days 4 and 7 after infection to analyze the levels of AeSNAP or ATPase and DENV2 by quantitative reverse transcription PCR (qRT-PCR).

### RNA extraction, cDNA synthesis and qRT-PCR-based assays

All mosquito RNA extractions were performed using TRIzol according to manufacturer’s protocol (Invitrogen, Carlsbad, CA). The RNA was subsequently used for production of a cDNA pool with iSCRIPT (BIORAD). The qRT-PCR assay was done using the iTaq kit according to the manufacturer’s instructions (BioRad). Oligos for the qRT-PCR reactions are shown in Table 2. Viral RNA or *Aedes* gene expression was normalized to Rp49 expression. Each sample was tested in quintuplicate for the *in vitro* studies.

### Statistical analysis

GraphPad Prism software was used to perform statistical analysis on all data. Transcription levels of DENV and *Aedes* candidates in mosquito cells, whole mosquito, were normalized using Rp49 housekeeping. The % of silencing efficacy was calculated following this formula: 100-(silenced gene*100/control (GFP)). Transcription levels were calculated using non -parametric Mann-Whitney Test, as indicated in the figure legends. Asterisk represents P value < 0.05.

## Results

### Identification of DENV binding proteins in *A*. *aegypti* salivary glands using a mass spectrometry assay

**Fig 1:**
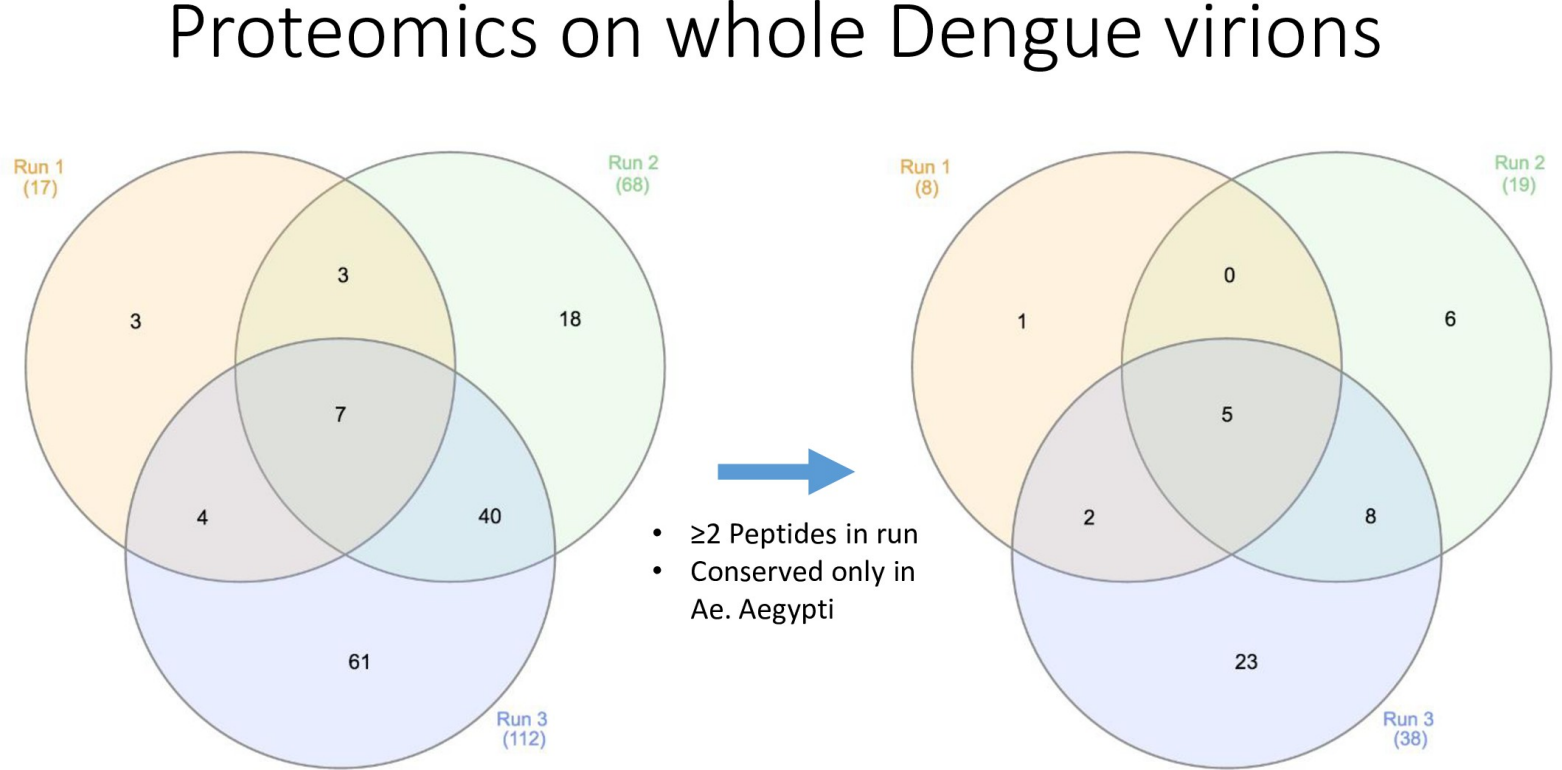
**Illustration of a three-cycle Venn diagram with the hits recovered from the mass spectrometry assay before selection (left) and after selection (right) of the hits**. Selection was based on the number of sequences found for every hit and their conservation in *A*. *aegypti*.

To identify *A*. *aegypti* salivary gland components that potentially interact with DENV virions, we utilized gradient sucrose purification of DENV virions that were pre-incubated with mosquito salivary gland extracts (SGE). DENV virions alone were used as control, allowing us to identify vector peptides that were only detected in samples containing SGE. In order to further eliminate proteins that possibly associated with DENV virions during propagation in C6/36 cells, which are derived from *A*. *albopictus* mosquitoes, we used the National Center for Biotechnology Information bioinformatic search database (BLASTp), to identify peptides that were conserved in *A*. *aegypti*, but not *A*. *albopictus*. This resulted in 45 *A*. *aegypti* salivary gland proteins that potentially interact with DENV virions. A list of these putative DENV binders obtained in three different runs was assembled (Table 1) and a Venn diagram was generated displaying the number of hits in each biological replicate and the overlap between the three runs (Fig 1). Eight, 19, and 38 proteins were found in runs 1, 2 and 3 respectively. We identified two unique proteins in only the 1^st^ and the 3^rd^ runs, eight unique proteins in only the 2^nd^ and the 3^rd^ runs and no overlapping proteins in only the 1^st^ and 2^nd^ runs. Finally, we identified five unique proteins in all three runs. The subset of the proteins which overlapped in multiple runs were then analyzed in subsequent experiments for their effect on DENV infection.

### Silencing genes which encode salivary gland proteins associated with virions, alters DENV infection in a mosquito cell line

**Fig 2:**
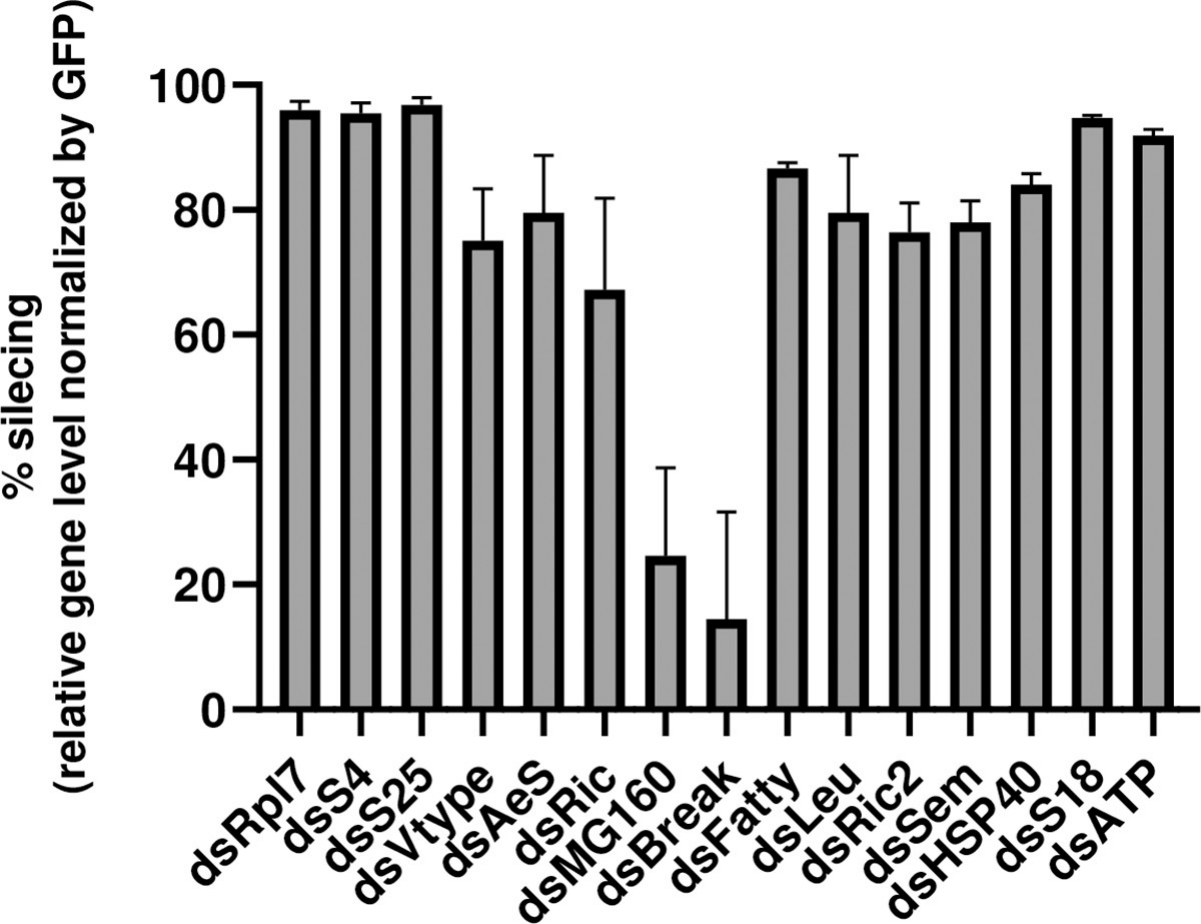
dsRNA silencing efficacy of *A*. *aegypti* genes in Aag2 cells. Hits from at least two experiments listed in Table 1 were knocked down in Aag2 cells using RNAi. At 48 h post-knockdown, silencing efficiency was analyzed by qRT-PCR, obtaining the relative levels of the specific gene normalized by Rp49 as housekeeping. Data is displayed as knockdown percentage of every hit compared to control (dsGFP). qRT-PCR analysis was done in pentaplicate, and the percentage of silencing was obtained comparing mean values of the relative gene levels between the specific genes and the GFP control (100%). Standard deviations are shown.

To elucidate if any of the protein candidates obtained from our mass spectrometry analysis (shown in Table 1) modified DENV infection, we used RNAi to reduce gene expression and analyzed the effect on viral infection. dsRNAs were generated against the genes encoding proteins that were found in at least 2 runs of the mass spectrometry analysis and used to silence these genes in an *Ae*. *aegypti* cell line, Aag2. A reduction between 75% and 95% in the mRNA transcripts was achieved at 48 hours post transfection in the genes screened, analyzed by qRT-PCR, with two exceptions (MG160 and Break), which were removed for further analysis (Fig 2).

**Fig 3:**
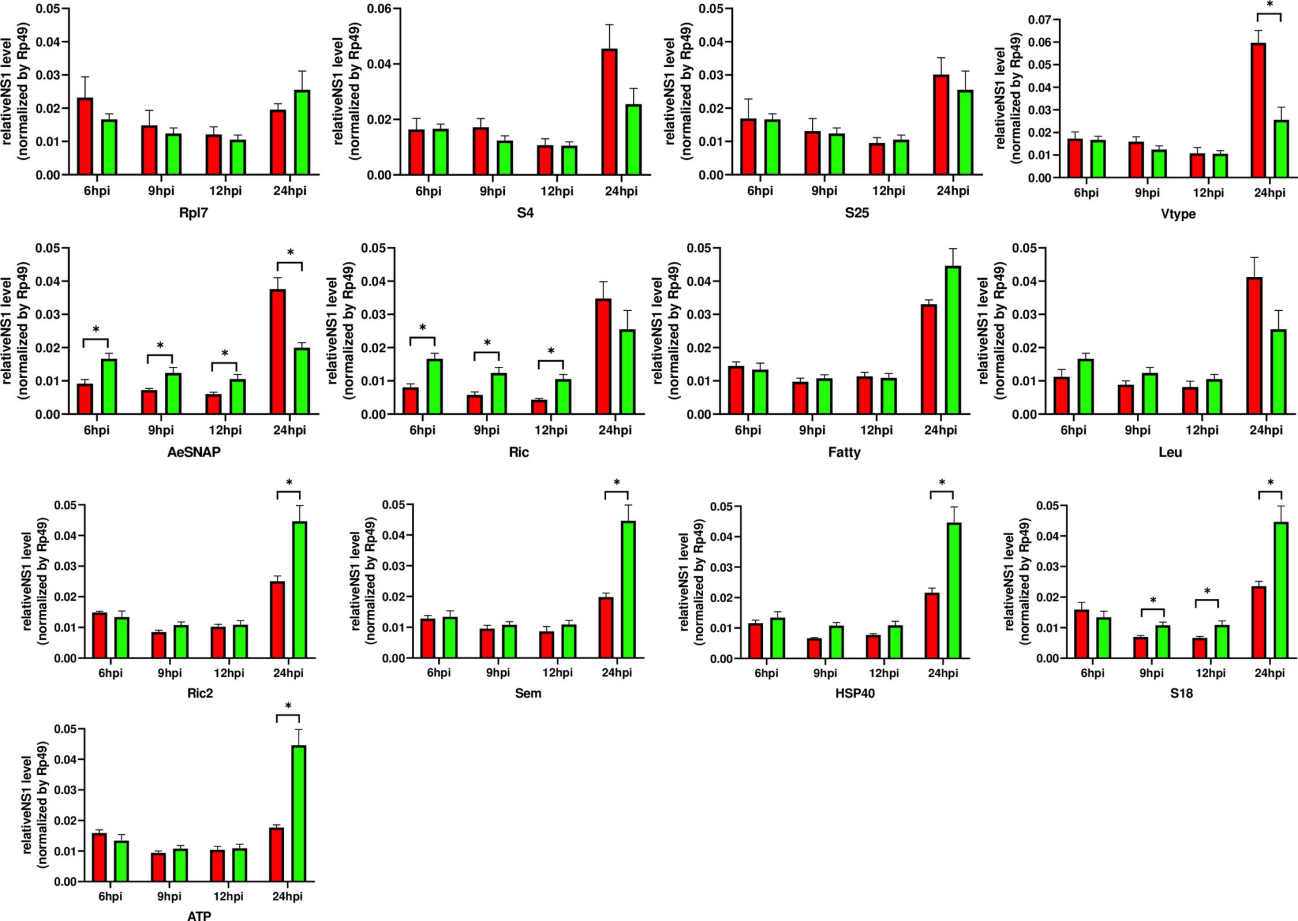
DENV infection relative levels in Aag2 cells. Viral burden was analyzed in Aag2 cells infected with DENV2 (MOI of 1.0) and was measured using qRT-PCR analysis at the timepoints indicated. Samples were taken at 6, 9, 12 and 24h post-knockdown to see the effect of silencing during DENV2 infection. The results represent the averages from samples done in pentaplicate, with the mean and standard deviation. In green, GFP-silenced control cells. In red, protein-silenced cells. Asterisks represent significant difference between samples, calculated by the Mann-Whitney nonparametric test (P < 0.05).

To identify the effect of protein knockdown on DENV infection, Aag2 cells were transfected with specific dsRNAs and 48 hours later were infected with DENV2 (MOI of 1.0). Each sample was then analyzed for intracellular viral production using qRT-PCR at 6, 9, 12 and 24 h post-infection (Fig 3). Knockdown of genes encoding several proteins led to significant changes in the intracellular viral load. At 6, 9 and 12 h post-infection, DENV titer was reduced in AeSNAP and Ric silenced cells as well as S18 at 9 and 12 hpi and Ric2, Sem, HSP40 and ATPase expression levels at 24 hpi. In contrast, we observed a significant increase in the DENV viral burden at 24 hpi for Vtype and surprisingly AeSNAP silenced cells (Fig 3).

### Silencing AeSNAP and ATP proteins alters DENV dissemination in *A*. *aegypti* mosquitoes

**Fig 4:**
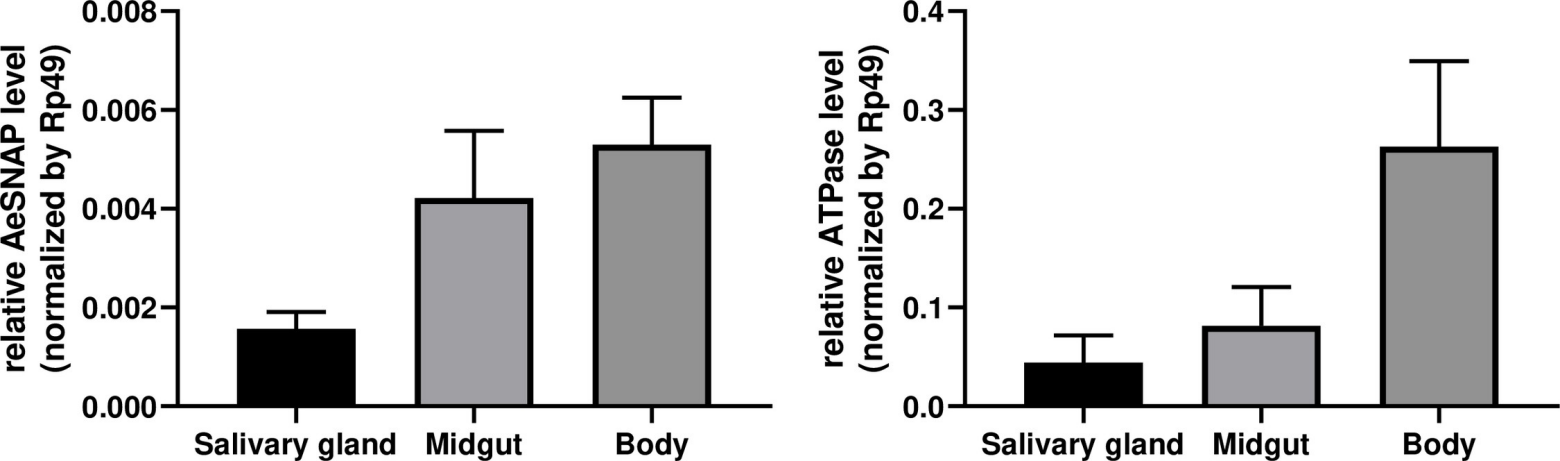
Differential gene expression of AeSNAP and ATPase. AeSNAP (left) and ATPase (right) relative expression was detected in salivary glands and midgut, and relative expression was also evaluated in the whole mosquito body in. AeSNAP and ATPase RNA levels were analyzed by qRT-PCR and normalized to the levels of Rp49.

After the *in vitro* analysis, we focused on two of the vector proteins that showed the greatest ability to alter viral replication, AeSNAP and ATPase proteins. Soluble N-ethylmaleimide-Sensitive Factor Attachment Proteins (SNAP) belong to a family of membrane proteins that have been implicated as the conserved core protein machinery required for all intracellular membrane fusion events that mediate intracellular trafficking [22]. The ATPase protein belongs to the calcium transporter ATPase pumps (Ca2+-ATPase or SERCA), membrane transport proteins ubiquitously found in the endoplasmic reticulum (ER) of all eukaryotic cells and enable a vast array of signaling pathways and physiological processes. [23]. Therefore, we confirmed the ubiquitous expression of these two candidates in the mosquito, analyzing salivary gland, midgut and the entire body (Fig 4).

**Fig 5:**
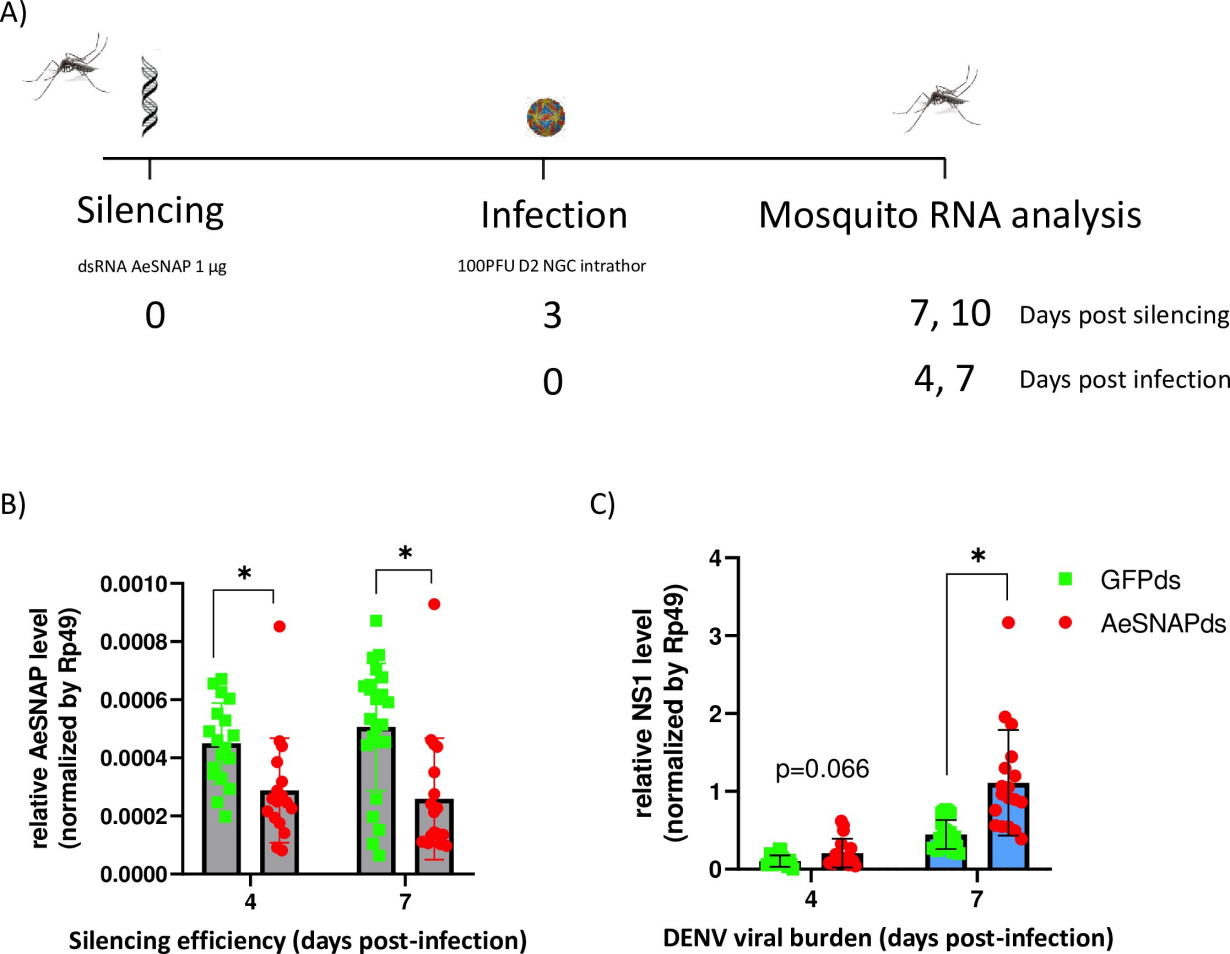
Dissemination analysis of DENV2 in AeSNAP dsRNA-knockdown mosquitoes. (A) Scheme of the strategy for dissemination studies in the *Aedes* mosquito. *A*. *aegypti* mosquitoes were intrathoracically injected with AeSNAP dsRNA, and at 72h, they were infected with 100PFU of DENV2 using the same route. Silencing efficacy and viral burden were evaluated at 4- and 7- day post infection. B) AeSNAP silencing efficacy (grey bars). (C) DENV2 viral load recovered from DENV2 infected *A*. *aegypti* mosquitoes (blue bars). AeSNAP and DENV2 RNA levels were analyzed by qRT-PCR and normalized to the levels of Rp49. Green squares correspond to GFP silenced mosquitoes (control) and red circles correspond with AeSNAP silenced mosquitoes. Results are representative of two independent experiments. Asterisks represent significant difference between samples, calculated by Mann-Whitney non-parametric test (p≤0.05).

**Fig 6:**
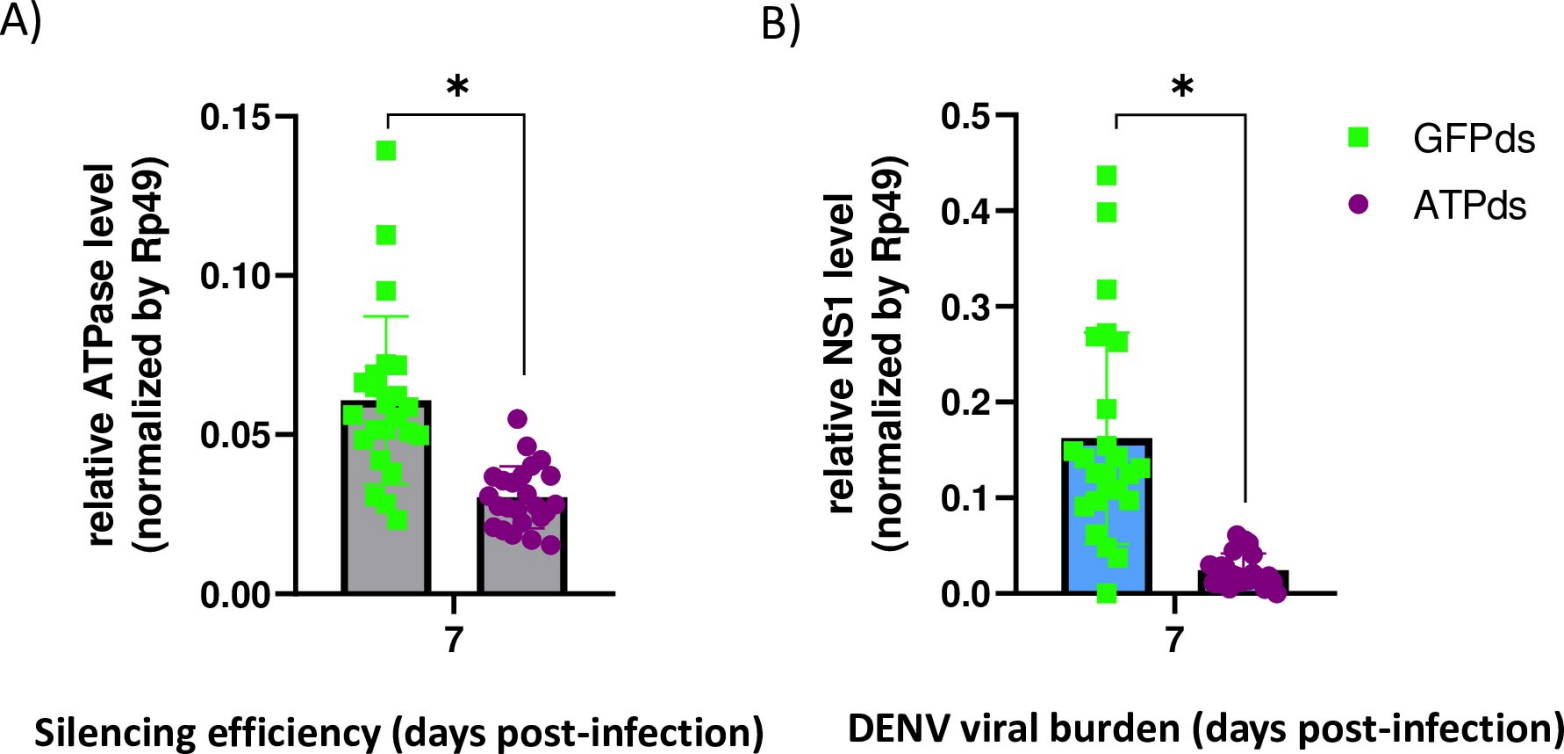
Dissemination analysis of DENV2 in ATPase dsRNA-knockdown mosquitoes. A) ATPase silencing efficacy (grey bars). B) DENV2 viral load recovered from DENV2 infected *A*. *aegypti* mosquitoes (blue bars). ATPase and DENV2 RNA levels were analyzed by qRT-PCR and normalized to the levels of Rp49. Green squares correspond to GFP silenced mosquitoes (control) and purple circles correspond with ATPase silenced mosquitoes. Results are representative of two independent experiments. Asterisks represent significant difference between samples, calculated by Mann-Whitney non-parametric test (p≤0.05).

Since both AeSNAP and ATPase are ubiquitously expressed in the entire body of the *A*. *aegypti* mosquito, we assessed the effect of these proteins in whole mosquitoes to attempt to identify any possible effect *in vivo* during DENV dissemination. For this aim, we intrathoracically injected *A*. *aegypti* mosquitoes with one μg of AeSNAP or ATPase dsRNA, and, 72 hours post injection, injected these same mosquitoes intrathoracically again with 100 PFU of DENV2. All mosquito infection experiments were performed by intrathoracic injections. This methodology has been widely used to better control the amount of infectious particles in the mosquito. In addition, it allows us to bypass the midgut barrier. DENV titer was then analyzed at 4 or 7 dpi (7 and 10 day post dsRNA injection, respectively) (Fig 5A). To confirm AeSNAP and ATPase gene knockdown, silencing efficiency was tested at the previously mentioned timepoints (4 and 7 dpi), and a significant reduction in the AeSNAP RNA transcript level (red) compared to the GFP control group (green) was observed (Fig 5B). Finally, DENV viral burden was analyzed, observing a tendency in viral burden increase at 4 dpi and a significant increase in the AeSNAP knockdown group (red) at day 7 post-infection. (Fig 5C). In addition, we also analyzed the role of ATPase during DENV dissemination. *Aedes* mosquitoes were silenced with ATP dsRNA (purple), and the silencing efficacy was confirmed at 7 dpi (10 day post dsRNA injection) (Fig 6A). Finally, viral burden was also measured at 7 day post infection (10 day post dsRNA injection), observing a significant reduction in DENV titers (Fig 6B). These results show that AeSNAP and ATPase proteins are involved in DENV dissemination control in the *Aedes aegypti* mosquito vector.

## Discussion

Infectious diseases transmitted by arthropod vectors, especially by mosquitoes, have acquired increasing medical importance over the few last decades. Among arthropod-borne viral infections, Dengue virus (DENV) is the most prevalent: more than 3.9 billion people in over 129 countries are at risk of contracting dengue, with an estimated 96 million symptomatic cases and an estimated 40,000 deaths every year (World Health Organization, Vector borne diseases). Although a DENV vaccine was just recently licensed by the U.S. Food and Drug Administration for the first time ever, it is far from ideal and its use is restricted only to seropositive individuals, due to the excess risk of severe dengue in seronegative vaccinees [24], where sub-optimal immunogenicity in first immune response to dengue predisposes them to a higher risk of severe disease when they experience their first natural dengue infection (ADE phenomena).

Other approaches, including insect vector control and blocking pathogen transmission within these vectors, are promising tools to control the spread of DENV [25]. To achieve this goal, it is necessary to understand the molecular mechanisms underlying the interactions between DENV and proteins in the *A*. *aegypti* mosquito. Success in prevention of pathogen transmission will primarily be based on targeting mosquito proteins which confer resistance or facilitates the infection within the vector. Recent studies have begun to define how the dengue viral proteins interact with host proteins to mediate viral replication and pathogenesis. A combined analysis of these studies, however, suggests that many virus-host protein interactions remain to be identified, especially for the mosquito host [26]. *Colpitts et al*., identified a mosquito-dengue protein interaction between NS2A and myelin protein expression factor (AAEL003670), observing a reduction of DENV and WNV infection in insect cells when the function of this mosquito protein was blocked [27]. To systematically analyze potential mosquito proteins which interact with DENV particles and could have a role during viral infection, we performed a mass spectrometry assay using purified DENV2 particles and *A*. *aegypti* salivary gland extracts. We identified a set of *A*. *aegypti* salivary gland proteins which potentially interact with the DENV virions. After this initial screening, we performed studies of silencing expression by RNAi in selected targets found in the mass spectrometry assay, both *in vitro* and *in vivo*. We demonstrated that two of these proteins, which are ubiquitously expressed in the *Aedes* mosquito, a synaptosomal-associated protein (AeSNAP) and a calcium transporter ATPase protein (ATPase), are involved in DENV viral burden regulation *in vivo*.

AeSNAP belongs to the SNAP family, which are implicated in intra-cellular trafficking and controlling a series of vesicle fusion events [28]. These proteins are regulators of vesicle trafficking in synaptic transmission [29], and have additional functions in autophagy and other endocytic and exocytic trafficking processes [30,31]. Moreover, the capsid phosphoprotein P of human para influenza virus type 3 (HPIV3) binds SNARE domains in SNAP29 protein, preventing binding of SNAP29 with SYX17, and hindering the formation of the ternary SNARE complex with VAMP8, required for autophagosome degradation [32,33]. SNAP29 binds the non-structural protein 2BC of the enterovirus-A71 (EV-A71), stimulating autophagy for its replication [34]. We show here that RNA interference-mediated knock-down of AeSNAP, an *A aegypti* mosquito protein, leads to an increase in the DENV viral burden in an *A*. *aegypti* cell line at 24 hpi and also in the whole organism *in vivo*. Surprisingly, in the *in vitro* analysis, we observed a significant decrease in the viral burden at early times post infection (6, 9 and 12 hpi), although this early reduction in the viral burden decreased gradually from 6 to 12 hour post-infection. This phenomenon could be explained by AeSNAP acting at multiple stages of the viral life cycle, showing both an antiviral (late) and a proviral (early times) behaviors, as it has been described for viperin or adenosine deaminases acting on RNA proteins (ADARs) (reviewed in [35,36]), although this fact should be further explored. This increase in the DENV viral burden under AeSNAP knockdown expression is consistent with the study previously mentioned, in which the HPIV3 capsid phosphoprotein P binds SNAP29, blocking autophagosome degradation and increasing virus production [32] and also with other studies focused on SNAREs and viral burden. For example, Ren et. al showed that inhibition of syntaxin 17 expression by specific small interfering RNAs resulted in an elevated amount of intracellular retained viral particles which facilitated the release of HCV virions by impairing of autophagosome-lysosome fusion [37].

We also identified an *A*. *aegypti* calcium transporter ATPase protein, ATPase, in our mass spectrometry assay. Calcium transporter ATPase proteins of the sarco (endo) plasmic reticulum (SERCA), the plasma membrane (PMCA), and the secretory pathway (SPCA) are crucial for muscle function, calcium cell signaling, calcium transport into secretory vesicles, mitochondrial function, and cell death [38–40]. Several viruses regulate host cell calcium concentrations in the cytoplasm and mitochondria, allowing viral gene expression and replication. For instance, a recent study performed in the human HAP-1 cell line revealed how that measles virus (MV), West Nile virus (WNV), Zika virus (ZIKV), Chikungunya virus (CHIKV), and also DENV use the host calcium pump secretory pathway calcium ATPase 1 (SPCA1) for calcium loading into the trans Golgi network, activating glycosyl transferases and proteases and then allowing viral maturation and spreading [41]. In our study, we found that the knockdown of this calcium transporter ATPase protein strongly reduced DENV burden in both the *A*. *aegypti* cell line and the *A*. *aegypti* mosquito, demonstrating a significant positive association between the level of ATPase protein and DENV viral burden. This finding is in line with another study, in which Vero cells treated with the SERCA-specific inhibitor Thapsigargin showed a significantly reduced level of viral replication for Peste des petits ruminants virus (PPRV) and Newcastle disease virus (NDV) [42].

Viruses are small intracellular parasites and rely on protein interactions to produce progeny inside host cells and to spread from cell to cell [43]. Viruses hijack a vast number of host factors to rewire cellular pathways and carry out processes required for replication. This is often achieved through physical interactions between viral and host proteins [44]. Understanding virus-host protein interactions in the mosquito vector can shed light on viral replication and resistance mechanisms. Furthermore, it could lead to important clinical translations, including the development of new therapeutic and vaccination strategies. In this study, we used a mass spectrometry screening assay to characterize a diverse group of mosquito proteins that are potentially associated with DENV virions, and characterized two of these, a synaptosomal-associated protein (AeSNAP) and a calcium transporter ATPase (ATPase) protein, in greater detail *in vivo*. We show that AeSNAP participates in DENV infection control, as its inhibition by RNAi led to a higher viral burden, whereas ATPase seems to be required for DENV infection in both the Aag2 mosquito cell line and in the *A*. *aegypti* mosquito vector. Further studies are needed in order to identify the specific pathways in which these two proteins are involved, and how they are mechanistically involved with DENV regulation, as well as to analyze other candidates (such as Vtype, Ric) described in the mass spectrometry list that were observed to alter DENV viral burden *in vitro* to a lesser degree. In addition, testing salivary gland viral loads would be another interesting goal to approach, to better analyze how these proteins affect viral transmission. Finally, this study suggests that these techniques can be used to examine interactions between other microbes and components of arthropod saliva. The identified components have the potential to serve as targets for preventing pathogen dissemination in the vector or the transmission to the vertebrate host.
